# Does Increasing the Minimum School-Leaving Age Affect the Intergenerational Transmission of Education? Evidence from Four European Countries

**DOI:** 10.1093/esr/jcab065

**Published:** 2021-12-22

**Authors:** Michael Grätz

**Affiliations:** Institut Des Sciences Sociales, University of Lausanne, 1015 Lausanne, Switzerland; Swedish Institute for Social Research (SOFI), Stockholm University, 10691 Stockholm, Sweden

## Abstract

Reforms in the minimum school-leaving age are candidates for policies that affect the intergenerational transmission of education. I propose that the societal contexts in which these reforms occur may moderate their effects on educational mobility. To test this hypothesis, I estimate the cross-country variation in the effects of increases in the minimum school-leaving age on educational mobility in four European countries. I employ a regression discontinuity design and data from the European Social Survey and the Survey of Health, Ageing and Retirement in Europe on Austria, Denmark, France, and the Netherlands. The findings provide no evidence to the hypothesis that the reforms in the minimum school-leaving age changed the association between the education of parents and the education of their children in any of the four countries. These findings are robust to measuring educational attainment in a multitude of ways, and they do not vary between men and women. The results are at odds with rational choice theories that expect reforms in the minimum school-leaving age to increase educational mobility.

## Introduction

Even in advanced, industrialized Western societies, men and women from different socioeconomic backgrounds do not have the same chances of achieving a high level of education (Breen and Jonsson, 2005; Torche, 2015). Notwithstanding the existence of educational inequalities in contemporary societies, there has been an increase in intergenerational educational mobility across cohorts in many advanced, industrialized Western societies (Breen *et al.*, 2009). These findings lead to the question of what caused this increase in educational mobility.^1^ Answering this question is important for designing policies that can reduce educational inequalities, which is a policy aim shared by many parties across the political spectrum.

Sociologists have noted that declines in educational inequalities have coincided with the process of educational expansion in many Western societies (Hout, 1988; Breen and Jonsson, 2007; Ballarino *et al.*, 2009; Breen, 2010; Pfeffer and Hertel, 2015; Bloome, Dyer and Zhou, 2018; Pöyliö, Erola and Kilpi-Jakonen, 2018). Research describing the simultaneous evolution of educational expansion and changes in inequality of educational opportunity, while being suggestive, can, however, not identify the causal relations between these two processes, as there are unobserved variables that can confound the relationship between educational expansion and educational mobility. To identify the causal effects of educational expansion on educational mobility, research designs are needed that can identify the effect of an increase in educational attainment on the association between social origin and educational attainment while controlling for the influence of unobserved variables.

Educational reforms that change the minimum school-leaving age can be employed as natural experiments for such a purpose.^2^ Four previous studies investigated the effects of increases in the minimum school-leaving age on intergenerational educational mobility in England and Wales (Sturgis and Buscha, 2015), West Germany (Betthäuser, 2017), and the United States (Rauscher, 2014, 2016). These studies led to diverging results. A decrease in the association between fathers’ occupation and children's school attendance was found in the United States (Rauscher, 2014, 2016). Educational mobility increased for children with a medium level of parental occupation compared to children with a high level of parental occupation but remained unchanged for children with a low level of parental occupation compared to children with a high level of parental occupation in West Germany (Betthäuser, 2017). In England and Wales, educational mobility at the level of compulsory education increased, but educational mobility remained unchanged at other levels of education (Sturgis and Buscha, 2015).

Differences across studies could be caused by the institutional setups of societies moderating the effects of reforms in the minimum school-leaving age on educational mobility. Differences in findings could, however, also be due to differences in methodological approaches and operationalizations of variables used in different studies. To discriminate between these two possibilities, I estimate the effects of reforms in the minimum school-leaving age on intergenerational mobility in four European countries using the same data and analytic strategy.

I analyse, using a regression discontinuity design (RDD), the effects of reforms that occurred in Austria, Denmark, France, and the Netherlands between 1959 and 1975. I apply the same methodological approach and operationalization of variables to pooled data from the European Social Survey (ESS) and the Survey of Health, Ageing and Retirement in Europe (SHARE). These data are particularly suited for comparative analyses, as they collect the same information for representative samples of respondents from different European countries. I select countries in which reforms in the minimum school-leaving age occurred during the period covered by my data and in which the reforms were implemented at the national level. In addition, I limit the case selection to reforms that increased the minimum school-leaving age but that did not introduce other large changes to the educational systems in the specific countries. This case selection criterion is necessary to isolate the causal effects of reforms in the minimum school-leaving age. Otherwise, these effects could be confounded by the effects of other changes in the educational systems, e.g. reforms in the allocation to tracks or in national curricula. Because of this later sample selection criterion, the number of countries included in this study is lower than that in studies that investigated the effects of reforms in the minimum school-leaving age on other outcomes (e.g. Braga, Checchi and Meschi, 2013; Schneeweis, Skirbekk and Winter-Ebmer, 2014; Fort, Schneeweis and Winter-Ebmer, 2016).

An important advantage of the comparative approach advanced in this study is that it allows me to increase the generalizability of the findings. Generalizability is a common problem that occurs if one natural experiment is studied in isolation (Torche, 2015). The findings from several cases provide a more robust case on which to draw general conclusions from policy reforms than does the analysis of a single case. For this reason, the methodological contribution of this article goes beyond the question of whether changes in the minimum school-leaving age affect the intergenerational transmission of education. It also proposes that analysing several natural experiments instead of a single one increases the external validity of analyses of the effects of political reforms on processes of sociological interest.

Analysing several reforms also allows me to test a specific hypothesis about cross-country variation in the effects of reforms in the minimum school-leaving age on educational mobility. I hypothesize that the effects of these reforms may be larger in contexts of low educational attainment prior to the implementation of the reforms.

The second contribution of my study is that I test two versions of rational choice theory (RCT): (i) a basic costs–benefits model and (ii) maximally maintained inequality (MMI) theory (Raftery and Hout, 1993). I argue that the basic costs–benefits model leads us to expect educational mobility to increase due to reforms in the minimum school-leaving age at all levels of education, while the second version of the RCT (MMI) expects educational mobility to increase due to the reforms only at levels of education, which are already fully attained by children from socioeconomically advantaged families. Previous research has not made this theoretical distinction and has therefore not tested these two predictions against each other.

## Does Increasing the Minimum School-Leaving Age Affect Intergenerational Educational Mobility? 

Education is an important predictor of life chances in contemporary societies (Heckman, 2000). Educational reforms are therefore an important topic in public debates. The most consequential educational reforms in Western societies have taken place in the last century, particularly in the period after the Second World War. Reforms in the minimum school-leaving age were arguably the most important drivers of educational expansion in Western countries in the 20th century. I focus on educational reforms that increased the minimum school-leaving age in four European countries between 1959 and 1975.

Why may reforms in the minimum school-leaving age affect the intergenerational transmission of education? Sturgis and Buscha (2015) speculated that a later minimum school-leaving age leads to an increase in educational mobility because children from socioeconomically advantaged families would have stayed in the educational system beyond the minimum school-leaving age even in the absence of any reform. Betthäuser (2017) developed this argument more extensively. His theory is based on the tradition of rational choice approaches to educational inequalities, according to which different social groups have different chances of succeeding in education (Boudon, 1973; Erikson and Jonsson, 1996; Breen and Goldthorpe, 1997). Changes in the minimum school-leaving age affect the educational decision-making of these groups, as they reduce the costs of completing a higher educational degree. This is the case because increasing the minimum school-leaving age while leaving the age needed to complete a certain degree unaffected shortens the time between the minimum school-leaving age and the completion of the degree.

In addition, changing the minimum school-leaving age may increase the success probabilities of children from socioeconomically disadvantaged families, as students who stay longer in the educational system can become better aware of their own abilities (Erikson and Jonsson, 1996). The influence of parents on educational decision-making may also decrease with the increasing age of children. Increasing the minimum school-leaving age may therefore lead to students making their own educational choices more often.

Another reason why increases in the minimum school-leaving age may reduce educational inequalities comes from research showing that inequalities in academic performance emerge in early childhood and are reduced by schools (Heckman, 2000; Raudenbush and Eschmann, 2015). Arguably the best causal evidence for this hypothesis comes from research showing that educational inequalities increase more in summer when no schooling takes place than during the school year (e.g. Downey, von Hippel and Broh, 2004; von Hippel, Workman and Downey, 2018; Holtmann and Bernardi, 2019). This perspective supports the notion that increasing the minimum school-leaving age reduces socioeconomic gaps in academic performance. Given that the equalizing effects of schools are rather small and that stronger equalizing effects of schools are expected for younger than for older children (Raudenbush and Eschmann, 2015; von Hippel, Workman and Downey, 2018), the effects of increasing the minimum school-leaving age on inequalities in educational achievement may, however, be rather weak. From these three perspectives, we can formulate, based on a basic costs–benefits model, hypothesis H1 as follows:

H1: Reforms in the minimum school-leaving age increase educational mobility at the level of education affected by the reform and at all following levels of education.

Are there any reasons why reforms in the minimum school-leaving age may not affect educational inequalities? This depends on which social group is affected most by these reforms. The arguments developed above assume either that all social groups are affected to the same degree by these reforms or that socioeconomically disadvantaged families are affected more than socioeconomically advantaged families. However, this does not necessarily have to be the case. What if the reforms mostly increased the educational attainment of low-performing children from socioeconomically advantaged families? This is precisely what Rauscher (2016) argued. She referred to Collins’ (1971) conflict theory, which stated that socioeconomically advantaged families obtained even more schooling if children from disadvantaged families increased their educational attainment. In line with these expectations, Triventi *et al.* (2020) argued that socioeconomically advantaged families find always ways to transmit their advantage to their children, independent of the education system. These theories lead to the opposite prediction than the theories motivating H1. We can therefore formulate hypothesis H2 as follows*:*

H2: Reforms in the minimum school-leaving age do not affect the intergenerational transmission of education.

Another important theory by which to understand the effects of reforms in the minimum school-leaving age on educational mobility could also be MMI theory (Raftery and Hout, 1993). This special form of RCT argues that a reduction in educational inequalities takes place only when the attainment of one level of education becomes almost universal for children from socioeconomically advantaged families. With respect to the effects of the reforms in the minimum school-leaving age on educational mobility, MMI theory makes two predictions, which together constitute *hypothesis H3.*

H3a: If an educational level has been almost universally attained by children from socioeconomically advantaged families, then educational mobility at this level of education will increase due to reforms in the minimum school-leaving age.

This can be due to a ceiling effect (Lucas, 2009), but it still implies an increase in educational mobility at this level of education.

H3b: At levels of education at which attainment by children from socioeconomically advantaged families is not almost universal, educational mobility remains unaffected by the reforms.

Testing H3a and H3b requires us to look at specific levels of educational attainment and to take into account the educational attainment of children from socioeconomically advantaged families at these levels of education prior to the reforms.

The expectations of MMI theory contrast with the expectations of the basic version of RCT, which motivates H1. The latter argues that increases in educational equality are due to reduced costs of completing a higher educational degree. Therefore, the basic costs-benefits model predicts increases in educational equality across all levels of education (H1). In contrast, MMI theory expects educational equality only to increase at levels of education, which are almost universally attained by children from socioeconomically advantaged families (H3a and H3b).

## Variation in the Effects of Reforms in the Minimum School-Leaving Age on Educational Mobility across Countries

Previous research has not investigated the cross-country variation in the effects of reforms in the minimum school-leaving age on educational mobility. The effects of these reforms may, however, vary as a function of the average level of educational attainment in a society prior to the reform. The reforms may increase educational mobility more in societies in which the average level of educational attainment is low at the time the reforms are implemented. With increasing educational attainment in a society, the gains of reforms in the minimum school-leaving age in terms of educational mobility may be reduced due to a ceiling effect. The descriptive statistics in Table 1 show that the average educational attainment in the pre-reform cohort was lower in France and in Austria than in Denmark and in the Netherlands. We can therefore formulate hypothesis H4, according to which:

**Table 1.** Overview of the reforms in the minimum school leaving age and the definition of cohorts

Country (year of reform, legal decision)	First birth year affected by the reform (actual implementation)	Increase in the minimum school leaving age, years	Before-reform cohort	After-reform cohort
Austria (1962)	1952	14–15	1944–1951	1953–1960
Denmark (1972)	1957	14–16	1949–1956	1958–1965
France (1959)	1953	14–16	1945–1952	1954–1961
Netherlands (1971/1975)	1957	14–16	1949–1956	1958–1965

H4: There are stronger effects of the reforms in the minimum school-leaving age on educational mobility in France and in Austria than in Denmark and in the Netherlands.

MMI theory can also be applied to explain cross-country variation in the effects of the reforms in the minimum school-leaving age on educational mobility. In some countries, a certain level of education may be almost universally attained by children from socioeconomically advantaged families, while this may not be the case in other countries. MMI theory would then expect educational mobility to increase only in the first set of countries. For this reason, the theory can potentially explain the cross-country variation in the effects of reforms in the minimum school-leaving age on educational mobility.

The findings from previous research are mixed but partly support the idea that the effects of reforms in the minimum school-leaving age on educational mobility are lower in countries with a high level of educational attainment compared to their counterparts. Sturgis and Buscha (2015) estimated the effects of the reform in England and Wales in 1972, which raised the minimum school-leaving age from 14 to 15 years, on socioeconomic inequalities in education. They estimated a multinomial logistic regression model comparing the attainment of five lower levels of education to the reference category of university education. They found that compared to university education, the reform changed socioeconomic inequalities only for one of these five lower levels of education, namely, for compulsory education. Children with a low or middle parental occupational status increased their likelihood of achieving compulsory education instead of university education compared to children with a high parental occupational status due to the reform. The authors did not report any results with respect to another reference category than university education. They interpreted the change in the way that children from parents with a low and with a medium occupational status achieved a higher level of education than before (compulsory education instead of less than compulsory education). The results, however, also implied that achieving university education became less likely compared to achieving a compulsory education for children from parents with a low or medium occupational status compared to children from parents with a high occupational status. Socioeconomic inequalities in education at the other levels of education were not affected by the reform in the minimum school-leaving age in England and Wales.

Betthäuser (2017) found that children from intermediate social classes improved their educational attainment compared to children from the most advantaged social class due to the increase in the minimum school-leaving age in West Germany. These reforms were carried out in different states in West Germany in different years between 1949 and 1969. The children from the most disadvantaged social class (unskilled workers) could not decrease their disadvantage in educational attainment compared to the children from the most advantaged social class through the reform. The findings of this study therefore provide some evidence for an increase in educational mobility but also demonstrate that the second most advantaged social class profited most from the reforms in the minimum school-leaving age in West Germany.

Rauscher (2014, 2016) found an increase in educational mobility attributable to the introduction of compulsory schooling laws in the United States in the 19th century. She found that the association between father’s occupational status and school attendance declined due to these laws. There were no non-linearities in the increase in educational mobility in the United States. It is difficult to compare Rauscher’s (2014, 2016) studies to others, as she studied reforms that introduced compulsory schooling while the other studies and the analysis reported in the present article estimate the effects of reforms that changed the minimum school-leaving age in countries in which compulsory schooling already existed.^3^

The results of these three studies are generally in line with the explanation of cross-country variation as a function of the average level of educational attainment in a society. There was only an unequivocal increase in educational mobility due to the introduction of compulsory schooling laws in the United States (Rauscher, 2014, 2016). The increases in the minimum school-leaving ages in societies with existing compulsory schooling laws had fewer effects on educational mobility (Sturgis and Buscha, 2015; Betthäuser, 2017). Because of the limited number of cases and the different analytic strategies used by these studies, it is difficult to verify my explanation of the cross-country variation in the effects of reforms in the minimum school-leaving age on educational mobility. To rule out the influence of methodological differences in study designs and to enlarge the number of cases, I estimate the cross-country variation in the effects of increases in the minimum school-leaving age on educational mobility using the same research design and data on four European countries.

Previous research did not test H3a and b (MMI theory) against H1 (the basic costs–benefits model) because these studies did either not report the educational attainment of children from socioeconomically advantaged families prior to the reforms in the minimum school-leaving age (Sturgis and Buscha, 2015; Betthäuser, 2017), or did they did not analyse specific levels of education (Rauscher, 2014, 2016).

## Data and Methods

### Data

The empirical analyses use data on four European countries from the ESS and the SHARE. The ESS samples the adult population in several European countries since 2001. I use data from the nine waves of the data currently available (European Social Survey, 2018). SHARE samples the population aged 50 years and older and their partners, who can be younger, in several European countries. I use data from the seven SHARE waves that are currently available (Börsch-Supan *et al.*, 2013; Börsch-Supan, 2019). SHARE is a panel dataset, but I include each individual only once, using the most recent information available for each variable. I pool observations from both datasets to increase the sample sizes underlying the analyses for each country, which allows me to obtain more precise estimates.^4^ To ensure that respondents could have been affected by the reforms, I limit the samples to respondents born in the country in which they were sampled.

The selection of reforms in the minimum school-leaving age in my study was based on two criteria. First, I only included reforms that affected respondents included in the data I use. Second, to isolate the effect of increasing the minimum school-leaving age, I excluded cases in which other, crucial changes to the education system happened simultaneously to the reforms in the minimum school-leaving age. This sample selection criterion leads, for instance, to the exclusion of a reform in the minimum school-leaving age in Denmark in 1958 from the analysis. This reform also changed the age at tracking and restructured the organization of middle school (Garrouste, 2010). I also excluded a reform in Italy in 1963, which also changed the age at tracking and the organization of secondary school in general (Fort, Schneeweis and Winter-Ebmer, 2016; van de Werfhorst, 2019). Finally, I excluded a reform in Sweden due to this sampling criterion. Meghir and Palme (2005) analysed an educational reform in Sweden that increased the minimum school-leaving age but that also affected tracking and changed the national curriculum (Garrouste, 2010). It is therefore not possible to attribute any effect of this reform to the change in the minimum school-leaving age.

Previous research has analysed reforms in West Germany (Betthäuser, 2017) and England and Wales (Sturgis and Buscha, 2015). However, the inclusion of these cases in my study was not possible. The reform in Germany was implemented in different states in different years and had to be excluded for this reason. Within the data I use, it is not possible to identify the state (*Bundesland*) where a respondent went to school. Furthermore, there may be cross-state variation in the effects of the reforms on educational mobility, which is overlooked by an analysis treating Germany as one case. In addition, the reform in England and Wales in 1972 could not be analysed because the UK did not participate in SHARE.

### Variables

#### Years of education

Respondents’ final educational attainment is measured as a continuous variable through years of education. The analysis of this outcome takes into account the whole distribution of education and not only specific levels of education. Both datasets provide information on the International Standard Classification of Education (ISCED) 1997 classification of final educational attainment. I recode this information into the ideal years of education needed to achieve the specific levels of education differentiated by the ISCED classification. The country-specific allocation of years of education to ISCED levels is based on information provided by Fort (2006: pp. 6–11).^5^

#### Compulsory education and post-secondary education

To test a basic costs–benefits model (H1) and MMI theory (H3) against each other, I present models with binary outcomes, which look at two specific levels of education. Ideally, I would look at educational transitions. This is, however, not possible, as only retrospective information on final educational attainment is available in the data. Therefore, I look at two binary dummy variables. The first dummy variable indicates the completion of compulsory schooling according to the length of schooling based on the minimum school-leaving age imposed by the country-specific reforms. In other words, this variable is coded as one if a respondent obtained at least the number of years of education she/he should if she/he complied with the reform. If the respondent had fewer years of education, she/he was coded as zero. I call this variable ‘compulsory education’. The second dummy variable, ‘postsecondary education’, is set to one if a respondent obtained a level of education corresponding to Categories 4–6 of the ISCED 1997 educational classification. These are the highest levels of education; in other words, the respondent completed either post-secondary non-tertiary (vocational) or post-secondary tertiary education. If that was not the case, the dummy was set to zero.

#### Social origin

I measure social origin using parental education, which is defined as the highest level of education of either parent with valid information. This practice follows the dominance approach (Erikson, 1984). I distinguish between a low level of education (no formal degree and ISCED 1 and 2), a medium level of education (ISCED 3 and 4), and a high level of education (ISCED 5 and 6). In all regression models, a medium level of parental education is the reference category. I use information on parental education as a measure of social origin because this information is available in both data sets (which is not the case for other measures of social origin such as parental occupation and parental income).^6^

#### Reform

I identify exposure to the reform based on respondents’ year of birth. I drop respondents who were in the first birth year affected by the reform because often only a part of a birth year was affected by a reform (Sturgis and Buscha, 2015). I define a dummy variable that is coded as one for all respondents who were born in the second birth year that was affected by the reform and the seven birth years after. I define the dummy variable as zero for all respondents who were born in the eight birth years immediately preceding the first birth year affected by the reform. All other respondents are dropped from the specific country samples.

The reform variable is defined using different birth years in different countries, as the reforms in the minimum school-leaving age were introduced in different years in different countries. Table 1 gives an overview of the reforms and the birth years included in the before- and after-reform cohorts. More details on the reforms and the rationale for defining the cohorts in the ways described in Table 1 are provided in the Supplementary Appendix A.

#### Gender

I control for gender through a dummy variable that is coded as one for men and zero otherwise. In addition, I demonstrate in a robustness check that the effects of the reforms in the minimum school-leaving age on educational mobility did not vary between women and men (Supplementary Table S9).

#### Country

I report separate results for the four countries included in the analysis. The descriptive statistics on the variables used in the analysis are reported in Table 2.

**Table 2.** Descriptive statistics by country

Variable	Austria	Denmark	France	Netherlands
Panel A: All				
Years of education	11.98 (2.83)	13.84 (2.51)	11.11 (3.72)	12.27 (2.54)
Compulsory education	0.85 (0.36)	0.97 (0.16)	0.73 (0.45)	0.96 (0.20)
Post-secondary education	0.26 (0.44)	0.50 (0.50)	0.28 (0.45)	0.36 (0.48)
Low parental education	0.36 (0.48)	0.35 (0.48)	0.70 (0.46)	0.72 (0.45)
Medium parental education	0.47 (0.50)	0.43 (0.50)	0.20 (0.40)	0.15 (0.35)
High parental education	0.17 (0.37)	0.22 (0.41)	0.10 (0.30)	0.14 (0.34)
Reform	0.50 (0.50)	0.48 (0.50)	0.49 (0.50)	0.43 (0.49)
Male	0.47 (0.50)	0.48 (0.50)	0.46 (0.50)	0.46 (0.50)
*N* (combined sample)	3,280	3,996	4,453	3,939
*N* (ESS)	1,432	1,994	2,446	2,453
*N* (SHARE)	1,848	2,002	2,007	1,486
Panel B: Before-reform cohort				
Years of education	11.79 (2.96)	13.70 (2.61)	10.79 (4.02)	12.12 (2.66)
Compulsory education	0.83 (0.38)	0.96 (0.20)	0.68 (0.47)	0.95 (0.23)
Post-secondary education	0.24 (0.43)	0.48 (0.50)	0.28 (0.45)	0.36 (0.48)
Low parental education	0.37 (0.48)	0.40 (0.49)	0.74 (0.44)	0.74 (0.44)
Medium parental education	0.45 (0.50)	0.43 (0.49)	0.17 (0.38)	0.14 (0.35)
High parental education	0.18 (0.38)	0.18 (0.38)	0.09 (0.29)	0.13 (0.33)
Male	0.46 (0.50)	0.50 (0.50)	0.47 (0.50)	0.47 (0.50)
*N*	1,626	2,061	2,272	2,261
Panel C: After-reform cohort				
Years of education	12.17 (2.68)	13.98 (2.39)	11.45 (3.36)	12.48 (2.35)
Compulsory education	0.87 (0.34)	0.99 (0.11)	0.78 (0.41)	0.98 (0.15)
Post-secondary education	0.28 (0.45)	0.52 (0.50)	0.28 (0.45)	0.37 (0.48)
Low parental education	0.36 (0.48)	0.30 (0.46)	0.65 (0.48)	0.69 (0.46)
Medium parental education	0.49 (0.50)	0.44 (0.50)	0.24 (0.43)	0.16 (0.36)
High parental education	0.15 (0.36)	0.26 (0.44)	0.11 (0.31)	0.15 (0.36)
Male	0.47 (0.50)	0.46 (0.50)	0.46 (0.50)	0.44 (0.50)
*N*	1,654	1,935	2,181	1,678

*Notes*: The table reports the means and in brackets the standard deviations.

*Sources*: ESS: Waves 1–9 and SHARE: Waves 1–7.

In addition to reporting descriptive statistics on the full sample, Table 2 reports descriptive statistics on the before- and after-reform cohorts. These descriptive statistics allow us to judge whether these cohorts differ on observed variables other than educational attainment. As seen from Table 2, this is not the case. This finding supports the notion that the central assumption of the analytic strategy, according to which the before- and the after-reform cohorts differ only in their levels of educational attainment, holds.

### Analytic Strategy

The causal effect of increasing the minimum school-leaving age on intergenerational educational mobility is identified by comparing the first cohort affected by the reform to the immediately preceding cohort. The difference in the association between parents’ and respondents’ education between the two cohorts is the estimate of the effect of the reform on relative educational mobility.

I estimate the following OLS (Ordinary Least Squares) regression models with respondents’ education Edu_C_, parental education Edu_P_, and the reform dummy Ref:

(1) $${\text{Edu}}_{C}=\alpha +\beta_{1}{\text{Edu}}_{P}+\beta_{2}\text{Ref}+\beta_{3}{\text{Edu}}_{P}X\;\text{Ref}+\beta_{4}t+\beta_{5}{\text{Edu}}_{P}X\;t+\beta_{i}V+\varepsilon$$

The interaction term Edu_P_ X Ref is the estimate of the causal effect of the specific educational reform on intergenerational educational mobility. I focus on this interaction term in the interpretation of the results. **V** is a vector of control variables, which includes a dummy for males, a dummy for the survey (ESS or SHARE), and a dummy for the survey wave in the case of the ESS. Importantly, I control for a linear time trend *t* and interactions between parental education and the linear time trend. Therefore, the analysis corresponds to an RDD.

To further motivate the empirical strategy, Supplementary Figures S1 and S2 report the variation in the average educational attainment at the population level (Supplementary Figure S1) and by parental education (Supplementary Figure S2) in all four countries. At least in France, there is evidence for an increase in educational attainment across years of birth in the pre- and post-reform cohorts, which is most pronounced for the offspring of parents with low education.

The identification of the effect of the reform relies on the assumption that the cohort immediately preceding the reform and the cohort immediately preceding the reform do not differ in any other aspect other than being exposed to the reform. To increase the plausibility of this assumption, it is important to limit the analysis to a short bandwidth around the discontinuity created by the reform, but there is a trade-off between a shorter bandwidth to increase identification and a larger bandwidth to increase the precision of estimates through more observations (Schneeweis, Skirbekk and Winter-Ebmer, 2014; Sturgis and Buscha, 2015).

The following analyses also have to assume that the Stable Unit Treatment Value Assumption (SUTVA) is not violated. SUTVA requires that the effect of the reform on the educational attainment of one respondent does not influence the educational attainment of other respondents (Gangl, 2010; Morgan and Winship, 2015). This assumption is plausible with respect to educational mobility. The educational attainment of those not affected by the reform is unlikely to have changed due to the reform. Supplementary Figures S1 and S2 also speak to this question. If there was anticipatory behaviour, we would expect an increase deviating from the linear time trend in the birth years preceding the reforms. In all countries, this is not the case. Therefore, SUTVA seems to hold.

To estimate binary outcomes, I employ Linear Probability Models (LPM). I employ LPMs because of the clear interpretation of their estimates and because they can be more easily compared across samples than logit and probit regression models (Angrist and Pischke, 2009; Mood, 2010; Auspurg and Hinz, 2011; Gomila, 2021). In addition, the main interest of my analysis is in interaction effects, which are easier to interpret in LPMs than in logit or probit regression models (Ai and Norton, 2003). The issue of heteroscedasticity is addressed by using robust standard errors.^7^

## Results

### Years of Education

I report the results separately for each country. I do not pool the data across countries as I want to test whether the reforms affected educational mobility within each country. I then compare these results across countries.

Table 3 reports the models estimating the effects of the reforms in the minimum school-leaving age on respondents’ years of education. Models 1 and 2 report the results for Austria. There are strong positive associations between parental education and children’s education in Austria. At the population level, children from low-educated parents have, on average, approximately 1.67 years of education less than children from medium-educated parents (the reference group). Children with highly educated parents have, on average, 1.24 years of education more than children with medium-educated parents (Model 1).

**Table 3.** OLS regression models estimating the effects of the reforms in the minimum school leaving age on years of education

Variable	Austria		Denmark		France		Netherlands	
	(1)	(2)	(3)	(4)	(5)	(6)	(7)	(8)
Low parental education	–1.67^*^ (0.19)	–1.61^*^ (0.22)	–1.18^*^ (0.16)	–1.20^*^ (0.18)	–2.87^*^ (0.27)	–2.75^*^ (0.30)	–1.30^*^ (0.20)	–1.29^*^ (0.23)
High parental education	1.24^*^ (0.24)	1.19^*^ (0.27)	1.41^*^ (0.20)	1.32^*^ (0.22)	2.04^*^ (0.40)	1.99^*^ (0.43)	1.61^*^ (0.27)	1.60^*^ (0.30)
Reform	0.17 (0.20)	0.11 (0.29)	0.07 (0.16)	0.20 (0.25)	–0.30 (0.22)	–0.62 (0.48)	–0.13 (0.16)	–0.17 (0.41)
Male	0.67^*^ (0.09)	0.67^*^ (0.09)	–0.41^*^ (0.07)	–0.41^*^ (0.07)	0.37^*^ (0.10)	0.38^*^ (0.10)	0.47^*^ (0.08)	0.47^*^ (0.08)
Low parental education × Reform		0.26 (0.44)		–0.11 (0.37)		0.49 (0.55)		0.06 (0.45)
High parental education × Reform		–0.26 (0.58)		–0.40 (0.42)		–0.32 (0.88)		–0.03 (0.61)
*N*	3,280	3,280	3,996	3,996	4,453	4,453	3,939	3,939

*Notes*: Standard errors in parentheses. All models control for the survey (ESS or SHARE), survey wave of the ESS, a linear time trend, and interactions between the linear time trend and parental education (estimates for these controls are not shown).

*Sources*: ESS: Waves 1–9 and SHARE: Waves 1–7.

* *P* < 0.05 (two-tailed tests).

The reform in the minimum school-leaving age in Austria had a small positive but statistically insignificant effect on years of education. There is no evidence that the reform did change the intergenerational transmission of advantage, as the interactions between the reform and parental education are substantively small and statistically insignificant (Model 2). For instance, the reform reduced the gap in educational attainment between children with low- and medium-educated parents from 1.61 to 1.61–0.26 = 1.35 years of education. This is a substantively small effect, which is also statistically insignificant and can therefore not be generalized from the analysed sample to the population level. As a result, the findings for Austria provide no support to H1 but are in line with H2.

Models 3 and 4 report the results for Denmark. On average, children with low-educated parents have 1.18 years of education less than children with medium-educated parents. Children with highly educated parents have, on average, 1.41 years of education more than children with medium-educated parents. The average effect of the reform in the minimum school-leaving age on educational attainment in Denmark was small and positive but, as in Austria, statistically insignificant (Model 3). There is no evidence of the reform changing the intergenerational transmission of advantage, as the interactions between parental education and the reform in the minimum school-leaving age are close to zero and statistically not significant (Model 4).

Models 5 and 6 report the results for France. In France, children with low-educated parents have, on average, 2.87 years of education less than children with medium-educated parents. Children with highly educated parents have, on average, 2.04 years of education more than children with medium-educated parents. There is no evidence that the reform in the minimum school-leaving age in France affected educational attainment (Model 5). There is also no evidence that the reform changed the difference in years of education between children with low- and children with medium-educated parents (Model 6). In line with H2, there was no statistically significant change in educational mobility in France.

Finally, Models 7 and 8 report the results for the Netherlands. On average, children with low-educated parents have 1.30 years of education less than children with medium-educated parents in the Netherlands. Children with highly educated parents have 1.61 years of education more, on average, than children with medium-educated parents. The results provide no support to the hypothesis of an effect of the reform in the minimum school-leaving age in the Netherlands on years of education (Model 7). In addition, no evidence for a change of increasing the minimum school-leaving on educational mobility is found in the empirical analysis. The two interaction terms are close to zero and statistically insignificant (Model 8). Therefore, the reform in the minimum schooling leaving age in the Netherlands had no clear, robust effect on the association between parental education and respondents’ years of education.

### Levels of Education

The models reported in Table 3 test how the reforms in the minimum school-leaving age affected the association between parental education and respondents’ years of education. The advantage of these models is that they take into account the whole distribution of education. To test the predictions of the basic costs–benefits model (H1) and of MMI theory (H3) against each other, it is, however, necessary to look at specific levels of education. I report models that estimate whether the respondents completed their compulsory education based on the length of compulsory schooling implemented by the reforms in the minimum school-leaving age and whether they obtained a post-secondary education.

Before presenting the results of these models, I first report the variation in the educational attainment of children in the pre-reform cohorts by parental education in Table 4. MMI theory expects educational mobility to increase only at levels of education that are almost completely attained by children from socioeconomically advantaged families prior to the reforms (H3). The basic costs–benefits model expects educational mobility to increase at the level of education affected by the reform in the minimum school-leaving age and at all higher levels of education (H1).

**Table 4.** Educational attainment in the pre-reform cohorts by parental education

Variable	Compulsory education (in %)	Post-secondary education (in %)
Panel A: Austria		
Low parental education	68.60	12.29
Medium parental education	90.56	22.16
High parental education	93.86	52.90
Panel B: Denmark		
Low parental education	92.15	32.88
Medium parental education	97.72	48.75
High parental education	98.91	78.26
Panel C: France		
Low parental education	60.27	19.30
Medium parental education	86.08	42.01
High parental education	92.68	72.68
Panel D: Netherlands		
Low parental education	93.21	27.21
Medium parental education	98.08	45.69
High parental education	98.59	75.97

*Sources*: ESS: Waves 1–9 and SHARE: Waves 1–7.

With respect to compulsory education, the attainment of children with medium and of children with highly educated parents was almost saturated in all countries prior to the reforms. Therefore, MMI theory predicts educational mobility to increase at this level of education in all countries (H3). The basic costs–benefits model (H1) also predicts educational mobility to increase at this level of education.

The attainment of post-secondary education was not saturated prior to the reforms, even not for children with highly educated parents. This pattern holds in all countries. Therefore, MMI theory predicts no change in educational mobility at this level of education in all countries (H3). The basic costs–benefits model (H1) expects, however, educational mobility to also increase at this level of education.

Table 5 presents LPMs that estimate the effects of the reforms in the minimum school-leaving age on compulsory education.

**Table 5.** Linear Probability Models estimating the effects of the reforms in the minimum school leaving age on compulsory education according to the minimum school leaving age implemented by the reforms

Variable	Austria		Denmark		France		Netherlands	
	(1)	(2)	(3)	(4)	(5)	(6)	(7)	(8)
Low parental education	–0.24^*^ (0.03)	–0.24^*^ (0.03)	–0.07^*^ (0.02)	–0.08^*^ (0.02)	–0.28^*^ (0.03)	–0.27^*^ (0.03)	–0.06^*^ (0.01)	–0.05^*^ (0.02)
High parental education	0.02 (0.02)	0.02 (0.03)	0.01 (0.01)	0.01 (0.01)	0.06 (0.03)	0.05 (0.04)	0.01 (0.01)	0.01 (0.02)
Reform	0.01 (0.03)	0.00 (0.03)	–0.00 (0.01)	0.00 (0.01)	–0.03 (0.03)	–0.07 (0.05)	–0.00 (0.01)	–0.01 (0.03)
Male	0.12^*^ (0.01)	0.12^*^ (0.01)	–0.01 (0.01)	–0.01 (0.01)	0.08^*^ (0.01)	0.09^*^ (0.01)	0.00 (0.01)	0.00 (0.01)
Low parental education × Reform		0.04 (0.06)		–0.01 (0.03)		0.06 (0.06)		0.01 (0.03)
High parental education × Reform		–0.01 (0.05)		–0.00 (0.01)		–0.06 (0.07)		0.01 (0.03)
*N*	3,280	3,280	3,996	3,996	4,453	4,453	3,939	3,939

*Notes*: Standard errors in parentheses. All models control for the survey (ESS or SHARE), survey wave of the ESS, a linear time trend, and interactions between the linear time trend and parental education (estimates for these controls are not shown).

*Sources*: ESS: Waves 1–9 and SHARE: Waves 1–7.

* *P* < 0.05 (two-tailed tests).

In Austria (Models 1 and 2), the probability of completing compulsory education was increased by four percentage points due to the reform for children from families with a low level of parental education (Model 2). For children from families with a medium level of parental education, there was no change in the probability of completing compulsory education. As a result, the association between parental education and the completion of compulsory education comparing children with parents with low education and children with parents with medium education was reduced from 0.24 before the reform to 0.24–0.04 = 0.20 percentage points after the reform. This change is substantively small and statistically insignificant.

In Denmark (Models 3 and 4), the difference in the completion of compulsory education between children with low and children with medium-educated parents increased from 0.08 to 0.08 + 0.01 = 0.09 percentage points due to the reform (Model 4). This substantively negligible and statistically insignificant decrease in educational mobility is in line with H2.

In France (Models 5 and 6), the reform decreased the association between parental education and compulsory education comparing children with low- to children with medium-educated parents from 0.27 prior to the reform to 0.27–0.06 = 0.21 after the reform (Model 6). The increase in educational mobility is statistically insignificant. The substantive size of the increase is also small.

In the Netherlands (Models 7 and 8), children with low-educated parents decreased the gap to children with medium-educated parents in their probability of completing compulsory education from 0.05 to 0.05–0.01 = 0.04. This decrease in educational mobility is statistically insignificant and substantively small.

These findings, which cannot rule out a zero effect of the reforms in the minimum school-leaving age on compulsory education, are not in line with the predictions of both the basic costs-benefits model (H1) and MMI theory (H3). In all countries, there is no evidence that the reforms affected educational mobility. The analysis of compulsory education therefore supports the findings with respect to years of education, according to which the hypothesis that the reforms in the minimum school-leaving age did not affect educational mobility (H2) cannot be ruled out.

The question arises whether the reforms in the minimum school-leaving age affected educational attainment beyond compulsory schooling. Table 6 reports LPMs estimating the effects of these reforms on respondents’ attainment of post-secondary (non-tertiary/vocational or tertiary) education.

**Table 6.** Linear Probability Models estimating the effects of the reforms in the minimum school leaving age on post-secondary education (Non-Tertiary or Tertiary, ISCED 1997 Levels 4–6)

Variable	Austria		Denmark		France		Netherlands	
	(1)	(2)	(3)	(4)	(5)	(6)	(7)	(8)
Low parental education	–0.10^*^ (0.03)	–0.10^*^ (0.03)	–0.18^*^ (0.03)	–0.20^*^ (0.04)	–0.24^*^ (0.04)	–0.22^*^ (0.04)	–0.19^*^ (0.04)	–0.18^*^ (0.05)
High parental education	0.28^*^ (0.04)	0.27^*^ (0.05)	0.30^*^ (0.04)	0.28^*^ (0.04)	0.32^*^ (0.05)	0.34^*^ (0.06)	0.35^*^ (0.05)	0.37^*^ (0.06)
Reform	0.02 (0.03)	0.03 (0.05)	0.02 (0.03)	0.07 (0.05)	–0.06^*^ (0.03)	–0.11 (0.07)	–0.04 (0.03)	–0.07 (0.09)
Male	0.04^*^ (0.01)	0.04^*^ (0.01)	–0.13^*^ (0.01)	–0.13^*^ (0.02)	–0.01 (0.01)	–0.00 (0.01)	0.08^*^ (0.01)	0.08^*^ (0.01)
Low parental education × Reform		0.01 (0.06)		–0.07 (0.08)		0.06 (0.07)		0.03 (0.09)
High parental education × Reform		–0.03 (0.11)		–0.09 (0.08)		0.07 (0.12)		0.07 (0.12)
*N*	3,280	3,280	3,996	3,996	4,453	4,453	3,939	3,939

*Notes*: Standard errors in parentheses. All models control for the survey (ESS or SHARE), survey wave of the ESS, a linear time trend, and interactions between the linear time trend and parental education (estimates for these controls are not shown).

*Sources*: ESS: Waves 1–9 and SHARE: Waves 1–7.

* *P* < 0.05 (two-tailed tests).

There is no evidence that the reforms in the minimum school-leaving age affected the intergenerational transmission of education at the level of post-secondary education. In all countries, the interactions between parental education and the reforms are not statistically significant (Models 2, 4, 6, and 8). Not only are the estimates statistically insignificant and go in different directions across countries, but they are also often substantively close to zero.

Given the imprecision of the estimates, it cannot be ruled out that the reforms had small effects on the associations between parental education and the respondents’ attainment of post-secondary education. It can, however, be ruled out that the reforms in the minimum school-leaving age had strong effects on educational mobility at this level of education. These findings are in line with MMI theory, which predicted no effect of the reforms on educational mobility with respect to post-secondary education, as attainment of this level of education was not saturated among children from socioeconomically advantaged families prior to the reforms (Table 4). However, the findings are also in line with hypothesis H2, which is also supported by the results for years of education and compulsory education. In sum, there is no evidence that the reforms in the minimum school-leaving age did affect educational mobility. These results therefore provide no support for H4, which predicted cross-country variation in the effects of the reforms on educational mobility.

### Robustness Check

In the analyses presented above, the causal effects of the reforms in the minimum school-leaving age on educational mobility are identified by looking at the change caused by the reform off a general time trend in each country. One concern with this identification strategy is that it may not control for a nonlinear, general trend towards higher educational attainment. To address this concern, I estimate models using a control case. As a control case, I select Switzerland for the following three reasons: (i) it is included in both ESS and SHARE; (ii) it is a comparable Western European democracy that is similar to Austria, Denmark, France, and the Netherlands; and (iii) it experienced no major educational reform during the time of the reforms in the minimum school-leaving age in the other four countries, at least not at the national level. The models using Switzerland as a control case are reported in Supplementary Table S8. To account for different distributions of years of education across countries, the outcome analysed in these models is years of education, which is standardized within each country. These models lead to virtually identical results. Therefore, this robustness check supports all the conclusions of this study.

I also test whether the effects of the reforms in the minimum school-leaving age on educational mobility differed between men and women. These models are reported in Supplementary Table S9. For both men and women, there is no evidence that the reforms affected the intergenerational transmission of education.

## Discussion and Conclusion

Do reforms in the minimum school-leaving age affect intergenerational mobility? Answers to this question vary across studies. Educational mobility increased due to reforms in the minimum school-leaving age in the United States (Rauscher, 2014, 2016). Increasing the minimum school-leaving age increased educational mobility for some but not for other groups in Germany (Betthäuser, 2017). At the same time, educational mobility was, with the exception of compulsory education, not affected by the reforms in the minimum school-leaving age carried out in England and Wales (Sturgis and Buscha, 2015).

How can these diverging results from previous research be interpreted? Differences in findings of effects of reforms in the minimum school-leaving age on intergenerational mobility between different studies could be due to methodological differences. This is precisely why I employed the same analytic strategy and data to compare the effects of four different reforms in the minimum school-leaving age on educational mobility across countries. On the other hand, differences in results across countries could be due to cross-country variation in the effects of reforms in the minimum school-leaving age on education mobility. In particular, I hypothesized that the level of educational attainment in a society prior to the reform would moderate the effects of the reforms on educational mobility. The findings from my study are, however, not in line with this hypothesis. In all four countries included in my analysis, no evidence was found that changing the minimum school-leaving age did affect educational mobility.

These results are at odds with some of the findings from previous research. Previous studies either did not control for any time trends (Sturgis and Buscha, 2015) or did only control for a general linear time trend but not for parental education-specific time trends (Rauscher, 2014, 2016; Betthäuser, 2017). Controlling for a parental education-specific time trend, i.e. by including interactions between the time trend and parental education, was crucial for my analyses, as doing so controls for the general increase in educational mobility across cohorts in the 20th century (Breen *et al.*, 2009). Therefore, not controlling for a parental education-specific time trend makes it more likely to attribute an increase in educational mobility wrongly to the reforms.

The findings of my empirical analyses are at odds with expectations based on RCT. A basic costs–benefits model expects increases in educational inequality due to reduced costs of completing a higher educational degree as a result of the reforms. This model therefore leads us to expect an increase in educational equality across all levels of education, including and following the level of education affected by the reforms. My empirical results provide no support to these predictions. My findings are also not in line with another version of RCT, i.e. MMI theory (Raftery and Hout, 1993). This theory predicts that educational mobility only increases due to reforms at levels of education, which are nearly completely attained by children from socioeconomically advantaged families prior to the reforms. However, in all four countries I studied, there is no evidence that the reforms affected compulsory or post-secondary education.

As a result, the findings of the present study can be brought in line with arguments according to which the intergenerational transmission of advantage is a process that, to a large extent, is unaffected by policy reforms (e.g. Clark, 2014). In addition, the findings are in line with conflict theories of education which claim that socioeconomically advantaged families respond to educational reforms in a way that allows them to continue to transmit their advantage to their offspring after the reforms were introduced (Collins, 1971; Rauscher, 2016; Triventi *et al.*, 2020).

Previous research employed only one measure of social origin, which in all cases was some measure of parental occupation (Rauscher, 2014, 2016; Sturgis and Buscha, 2015; Betthäuser, 2017). Contrary to these studies, I employed a measure of parental education. Different measures of social origin are highly but not completely collinear (Bukodi and Goldthorpe, 2013; Erola, Jalonen and Lehti, 2016; Mood, 2017). It would therefore be welcome to see whether the results are robust to employing different measures of social origin. Unfortunately, my data do not allow me to conduct a robustness check in this respect, as only one indicator of social origin (parental education) is available for a large enough number of respondents in my data.^8^

Theoretically, it would be possible that tracking could moderate the effect of the reforms in the minimum school-leaving age on educational mobility. At the time that the reforms in the minimum school-leaving age were conducted, all the countries I looked at tracked children into a more advanced academically oriented and a less advanced track in lower secondary school. This tracking occurred at ages 10 (Austria), 14 (Denmark), 11 (France), and 12 (Netherlands). Tracking in lower secondary school in Denmark and France was abolished only after the reforms in the minimum school-leaving age, which I analyse in the present study. Therefore, my study does not analyse a country in which tracking took place after the minimum school-leaving age stipulated by the reforms. For this reason, the present study can unfortunately not test whether tracking moderates the effects of the reforms in the minimum school-leaving age on educational mobility. An interesting hypothesis for further researchers to test is whether reforms in the minimum school-leaving age, which occur before the allocation to different tracks in secondary school, can increase educational mobility.

One may perceive as a limitation of my study that it focuses on educational reforms that occurred a long time ago. These reforms may not be representative of reforms carried out more recently. Nevertheless, it is instructive to study these reforms for at least three reasons. First, these reforms implied stronger changes to the educational system than those made by the reforms in the minimum school-leaving age that are carried out today in advanced, industrialized Western societies. The effects of the reforms included in my analysis are therefore likely to provide upper bound estimates of the effects of reforms in the minimum school-leaving age on educational mobility.

Second, the discussion on educational expansion is ongoing in Western societies. It has shifted, however, from the compulsory to the university level. Analyses of the effects of university reforms on intergenerational mobility are therefore an important extension of analyses of reforms in the minimum school-leaving age.

Third, some countries are still carrying out reforms in the minimum school-leaving age. This includes less-developed countries but also, for instance, the region of Geneva (Switzerland), which increased the minimum school-leaving age from 17 to 18 years in 2018. Such reforms may improve the overall level of educational attainment in a society, but my results suggest that these reforms are unlikely to affect educational mobility.

Finally, this article makes a methodological contribution to studies estimating the effects of policies on outcomes of sociological interest. To draw general conclusions from these studies, a cross-national approach, as advanced in this article, is needed. Studies of the effects of policies on intergenerational mobility usually focus on single cases. These single case studies have, however, the problem that their generalizability is unclear (Torche, 2015). Applying a cross-national, comparative approach is an important methodological advancement of this line of research. It promises to increase the external validity of estimates of the causal effects of policies on intergenerational mobility.

## Supplementary Material

jcab065_Supplementary_Data

## Footnotes

1 This study focuses on relative educational mobility, i.e. the association between parental and child education. This can be distinguished from absolute educational mobility, which analyses whether children achieve a higher (or lower) level of education than their parents. Relative educational mobility is also known as inequality of educational opportunity (Boudon, 1973). Throughout the manuscript, I use the terms educational mobility and inequality of educational opportunity interchangeably.

2 Minimum school-leaving age is the youngest age at which students can leave school. It therefore corresponds to the completion of compulsory education, and I use these two terms interchangeably.

3 Three of these four studies analyse not only educational but also occupational mobility (Sturgis and Buscha, 2015; Rauscher, 2016; Betthäuser, 2017). Given the potential violation of SUTVA in estimating the effects of reforms in the minimum school-leaving age on occupational mobility, I limit my analysis to the study of educational mobility. I therefore also focus on educational mobility in discussing the findings from previous research. The three studies also diverge in their findings with respect to intergenerational occupational mobility. Rauscher (2016) found that occupational mobility declined in the short term but did not change in the long term in the United States. Sturgis and Buscha (2015) found no effect of the reform in the minimum school-leaving age in England and Wales on occupational mobility. Betthäuser (2017) found similar effects of the reform in Germany on occupational mobility as he found on educational mobility.

4 The results of the separate analyses of ESS and SHARE are in line with those of the pooled data, but the estimates are less precisely estimated due to smaller sample sizes. For that reason, I am convinced that the interpretation should focus on the more precise estimates obtained using the pooled data. I report the models using only ESS or SHARE data in Supplementary Tables S1 and S2.

5 I test the robustness of the results to using self-reported years of education. The models using self-reported years of education as the outcome are reported in Supplementary Table S3. They lead to virtually identical estimates. In addition, I test the robustness of the results to using ordered logistic regression models with levels of education (ISCED categories) as the outcome variable. These models are reported in Supplementary Table S4. The models lead to the same results; the reforms did not affect educational mobility.

6 The number of respondents with missing information on parental education in both data sets is reported in Supplementary Table S7. The missing information may decrease the representativeness of the sample. However, the share of missing information varies across countries and data sets. Given the results are robust across countries and data sets, the missing information may not lead to large biases in the estimates.

7 Even though I prefer for the mentioned reasons the use of LPMs, I report in Supplementary Tables S5 and S6 logistic regression models. These models support all the conclusions derived from the LPMs.

8 For some respondents, information on fathers’ and mothers’ occupation during childhood was available. However, in both surveys, this information was not collected in all waves, and using this information would result in small sample sizes and selective samples. Other data are therefore needed to conduct an analysis comparing the effects of reforms in the minimum school-leaving age on the association between parental education and child education *and* the association between parental occupation and child education.

## Notes

**Michael Grätz** is a lecturer in sociology at the University of Lausanne, where he conducts a research project financed via an Ambizione grant of the Swiss National Science Foundation. He is also an associate professor (docent) at the Swedish Institute for Social Research (SOFI), Stockholm University. His research interests are in the fields of child development, social stratification, and social demography. A major aim of his research is to understand the intergenerational transmission of advantage.
