# Supplementary material for: Does Increasing the Minimum School-Leaving Age Affect the Intergenerational Transmission of Education? Evidence from Four European Countries
Source: Eur Sociol Rev. 2021 Dec 22;38(4):543–59. doi: 10.1093/esr/jcab065 (PMC9315953; doi:10.1093/esr/jcab065)
Supplement: jcab065_Supplementary_Data [file jcab065_supplementary_data.pdf]

ONLINE SUPPLEMENT TO

Does Increasing the Minimum School-Leaving Age Affect the Intergenerational Transmission of Education? Evidence from Four European Countries

Published in 2022 in the European Sociological Review

Michael Grätz

Institut des sciences sociales, University of Lausanne

Swedish Institute for Social Research (SOFI), Stockholm University

E-Mail: michael.gratz@unil.ch

This version: November 8, 2021

APPENDIX A. DETAILS ON THE EDUCATIONAL REFORMS

Austria. Austria passed a law to increase the minimum school leaving age by one year in 1962 (Fort et al. 2016; Gathmann et al. 2013; Schneeweis et al. 2014). The increase in the minimum school leaving age came into effect in 1966. The reform changed the minimum school leaving age from 14 to 15. The first cohort affected by the reform were children born in 1952 (Gathmann et al. 2015). The reform did not change the age at tracking, which was 10 (van de Werfhorst 2019). In other words, the allocation of children to more academically and more vocationally oriented tracks was not affected by the change in the minimum school leaving age, as the reform only led children to spend one year more in school after having been allocated to different tracks.

Denmark. Denmark introduced a policy to increase the minimum school leaving age by two years in 1971 (Brunello et al. 2009; Fort et al. 2016; Garrouste 2010; Gathmann et al. 2013; Schneeweis et al. 2014). Children who were born in 1957 were the first to be affected by the reform. Therefore, my analysis compares children born in 1958 and later to those born in 1956 and earlier. The age at tracking for the pre- and the post-reform cohorts in my data was 14 (van de Werfhorst 2019). The reform of the minimum school leaving age happened in isolation from other reforms (Fort 2006; Gathmann et al. 2013). More academically oriented grammar schools and more vocationally oriented tracks already existed before the reform.

France. The French reform in 1967 increased the minimum school leaving age by two years from age 14 to 16 (Grenet 2013; Fort et al. 2016; Schneeweis et al. 2014). The law was decided upon in 1959 but the reform was only implemented in 1967. Already before the reform in the minimum school leaving age, the French school system was divided into a more vocationally

oriented track (consisting of an extended primary school followed by vocational schools) and a more academically oriented track (consisting of *collège*, followed by *lycée*) (Grenet 2013). This tracking structure, as well as the age of tracking at 11, remained unchanged by the increase in the minimum school leaving age in 1967. The children, who were first affected by this reform, were born in 1953. This cohort is dropped from the analysis, which compares children born in 1954 and later to those born in 1952 and earlier.

Netherlands. Two reforms, which became laws in 1971 and 1975, increased the minimum school leaving age by one year each in the Netherlands (Fort et al. 2016). The total increase was therefore two years from age 14 to 16. The first cohort affected by the first reform was born in 1957 and the first cohort affected by the second reform in 1959. I drop the cohort born in 1957 from the analysis and compare those born in 1958 and later to those born in 1956 and earlier. Age at tracking in the Netherlands was at 12 years and did not change during the reform in the minimum school leaving age (van de Werfhorst 2019). The allocation to tracks happened before the minimum school leaving age, both before and after the reform.

REFERENCES

Brunello, G., Fort, M. and Weber, G. (2009). Changes in compulsory schooling, education, and the distribution of wages in Europe. *The Economic Journal,* **119**, 516–539.

Fort, M. (2006). Educational reforms across Europe: A toolbox for empirical research. Source: http://citeseerx.ist.psu.edu/viewdoc/download?doi=10.1.1.571.597&rep=rep1&type=pdf, last accessed March 22, 2020.

Fort, M., Schneeweis, N. and Winter-Ebmer, R. (2016). Is education always reducing fertility? Evidence from compulsory schooling reforms. *The Economic Journal,* **126**, 1823–1855.

Garrouste, C. (2010). 100 years of educational reforms in Europe: A contextual database. *JRC Scientific and Technical Reports*, No. 57357. Available at: http://publications.jrc.ec.europa.eu/repository/bitstream/JRC57357/reqno_jrc57357.pdf, last accessed March, 22, 2020.

Gathmann, C., Jürgens, H. and Reinhold, S. (2015). Compulsory schooling reforms, education mortality in twentieth century Europe. *Social Science and Medicine*, **127**, 74–82.

Grenet, J. (2013). Is extending compulsory schooling alone enough to raise earnings? Evidence from French and British compulsory schooling laws. *The Scandinavian Journal of Economics,* **115**, 176–210.

Schneeweis, N., Skirbekk, V. and Winter-Ebmer, R. (2014). Does education improve cognitive performance four decades after school completion? *Demography,* **51**, 619–643.

van de Werfhorst, H. G. (2019). Early tracking and social inequality in educational attainment: Educational reforms in 21 European countries. *American Journal of Education*, **126**, 65–99.

APPENDIX B. SUPPLEMENTARY ANALYSES

TABLES

Table S1. OLS Regression Models Estimating the Effects of the Reforms in the Minimum School Leaving Age on Years of Education, Using only Data from the European Social Survey (ESS)

	**Austria**		**Denmark**		**France**		**Netherlands**	
	(1)	(2)	(3)	(4)	(5)	(6)	(7)	(8)
Low parental education	–1.47*	–1.55*	–1.32*	–1.24*	–3.34*	–3.32*	–1.41*	–1.56*
	(0.25)	(0.29)	(0.23)	(0.26)	(0.35)	(0.39)	(0.28)	(0.31)
High parental education	1.69*	1.68*	1.92*	1.85*	2.21*	2.14*	2.00*	1.84*
	(0.37)	(0.42)	(0.29)	(0.31)	(0.53)	(0.59)	(0.35)	(0.40)
Reform	0.10	0.24	0.08	0.05	–0.09	–0.10	–0.36	0.19
	(0.27)	(0.37)	(0.25)	(0.39)	(0.29)	(0.63)	(0.21)	(0.55)
Male	0.47*	0.47*	–0.50*	–0.50*	0.40*	0.40*	0.56*	0.56*
	(0.12)	(0.12)	(0.11)	(0.11)	(0.14)	(0.14)	(0.10)	(0.10)
Reform X Low parental education		–0.35		0.37		0.06		–0.62
		(0.57)		(0.57)		(0.72)		(0.60)
Reform X High parental education		–0.04		–0.41		–0.32		–0.69
		(0.94)		(0.64)		(1.13)		(0.78)
N	1,432	1,432	1,994	1,994	2,446	2,446	2,453	2,453

Notes: Standard errors in parentheses. All models control for survey wave, a linear time trend, and interactions between the linear time trend and parental education (estimates for these controls are not shown).
Source: ESS, Waves 1–9.
* $p < 0.05$ (two-tailed tests)

Table S2. OLS Regression Models Estimating the Effects of the Reforms in the Minimum School Leaving Age on Years of Education, Using Only Data from the Survey of Health, Ageing and Retirement in Europe (SHARE)

	**Austria**		**Denmark**		**France**		**Netherlands**	
	(1)	(2)	(3)	(4)	(5)	(6)	(7)	(8)
Low parental education	–1.85*	–1.65*	–1.04*	–1.17*	–2.30*	–2.07*	–1.29*	–1.06*
	(0.28)	(0.32)	(0.21)	(0.24)	(0.41)	(0.45)	(0.30)	(0.35)
High parental education	1.06*	1.01*	0.96*	0.86*	2.01*	2.00*	0.98*	1.18*
	(0.32)	(0.36)	(0.27)	(0.31)	(0.59)	(0.64)	(0.42)	(0.49)
Reform	0.20	–0.04	0.07	0.32	–0.54	–1.27	0.21	–0.54
	(0.28)	(0.43)	(0.21)	(0.31)	(0.34)	(0.76)	(0.26)	(0.63)
Male	0.83*	0.84*	–0.32*	–0.33*	0.34*	0.35*	0.29*	0.29*
	(0.13)	(0.13)	(0.10)	(0.10)	(0.16)	(0.16)	(0.12)	(0.12)
Reform X Low parental education		0.80		–0.51		1.05		0.92
		(0.64)		(0.47)		(0.86)		(0.70)
Reform X High parental education		–0.25		–0.41		–0.17		0.82
		(0.76)		(0.56)		(1.38)		(1.00)
N	1,848	1,848	2,002	2,002	2,007	2,007	1,486	1,486

Notes: Standard errors in parentheses. All models control for a linear time trend, and interactions between the linear time trend and parental education (estimates for these controls are not shown).
Source: SHARE, Waves 1–7.
* $p < 0.05$ (two-tailed tests)

Table S3. OLS Regression Models Estimating the Effects of the Reforms in the Minimum School Leaving Age on Self-Reported Years of Education

	**Austria**		**Denmark**		**France**		**Netherlands**	
	(1)	(2)	(3)	(4)	(5)	(6)	(7)	(8)
Low parental education	–1.45*	–1.25*	–1.47*	–1.45*	–2.39*	–2.34*	–1.43*	–1.46*
	(0.24)	(0.28)	(0.19)	(0.21)	(0.23)	(0.25)	(0.22)	(0.25)
High parental education	0.91*	1.01*	1.70*	1.65*	1.84*	1.75*	1.46*	1.48*
	(0.30)	(0.34)	(0.24)	(0.27)	(0.33)	(0.37)	(0.29)	(0.32)
Reform	0.44	0.06	0.14	0.16	–0.27	–0.36	–0.09	–0.00
	(0.25)	(0.37)	(0.20)	(0.30)	(0.19)	(0.41)	(0.17)	(0.44)
Male	0.18	0.18	–0.23*	–0.22	0.04	0.04	0.62*	0.62*
	(0.11)	(0.11)	(0.09)	(0.09)	(0.09)	(0.09)	(0.08)	(0.08)
Low parental education X Reform		0.84		0.07		0.20		–0.14
		(0.55)		(0.45)		(0.47)		(0.49)
High parental education X Reform		0.46		–0.22		–0.47		0.07
		(0.73)		(0.51)		(0.74)		(0.66)
N	3,280	3,280	3,996	3,996	4,453	4,453	3,939	3,939

Notes: Standard errors in parentheses. All models control for the survey (ESS or SHARE), survey wave of the ESS, a linear time trend, and interactions between the linear time trend and parental education (estimates for these controls are not shown).
Sources: ESS, Waves 1–9 and SHARE, Waves 1–7.
* $p < 0.05$ (two-tailed tests)

Table S4. Ordered Logistic Regression Models Estimating the Effects of the Reforms in the Minimum School Leaving Age on Levels of Education (ISCED categories)

	**Austria**		**Denmark**		**France**		**Netherlands**	
	(1)	(2)	(3)	(4)	(5)	(6)	(7)	(8)
Low parental education	0.28*	0.30*	0.44*	0.43*	0.22*	0.23*	0.36*	0.36*
	(0.04)	(0.05)	(0.05)	(0.06)	(0.03)	(0.04)	(0.05)	(0.06)
High parental education	2.70*	2.59*	3.35*	2.97*	3.54*	3.58*	3.67*	3.53*
	(0.48)	(0.52)	(0.54)	(0.53)	(0.74)	(0.83)	(0.73)	(0.80)
Reform	1.11	1.06	1.08	1.25	0.86	0.72	0.91	0.93
	(0.17)	(0.23)	(0.14)	(0.24)	(0.10)	(0.18)	(0.11)	(0.28)
Male	1.65*	1.66*	0.74*	0.74*	1.27*	1.28*	1.48*	1.48*
	(0.12)	(0.12)	(0.04)	(0.04)	(0.07)	(0.07)	(0.09)	(0.09)
Low parental education X Reform		1.25		0.91		1.30		1.00
		(0.42)		(0.26)		(0.38)		(0.34)
High parental education X Reform		0.83		0.58		1.03		0.85
		(0.35)		(0.20)		(0.47)		(0.39)
N	3,280	3,280	3,553	3,553	4,453	4,453	3,939	3,939

Notes: The coefficients are reported as odds ratios. Standard errors in parentheses. All models control for the survey (ESS or SHARE), survey wave of the ESS, a linear time trend, and interactions between the linear time trend and parental education (estimates for these controls are not shown).
Sources: ESS, Waves 1–9 and SHARE, Waves 1–7.
* $p < 0.05$ (two-tailed tests)

Table S5. Logistic Regression Models Estimating the Effects of the Reforms in the Minimum School Leaving Age on Compulsory Education according to the Minimum School Leaving Age Implemented by the Reforms

	**Austria**		**Denmark**		**France**		**Netherlands**	
	(1)	(2)	(3)	(4)	(5)	(6)	(7)	(8)
Low parental education	0.19*	0.20*	0.26*	0.23*	0.24*	0.27*	0.33	0.38
	(0.04)	(0.05)	(0.09)	(0.10)	(0.05)	(0.06)	(0.19)	(0.24)
High parental education	1.35	1.35	1.73	1.68	1.66	1.33	1.42	1.65
	(0.50)	(0.58)	(1.29)	(1.43)	(0.63)	(0.54)	(1.24)	(1.63)
Reform	1.16	1.01	1.14	1.67	0.85	0.56	1.04	0.49
	(0.27)	(0.40)	(0.52)	(1.56)	(0.13)	(0.24)	(0.38)	(0.78)
Male	3.06*	3.06*	0.81	0.80	1.59*	1.60*	1.11	1.11
	(0.35)	(0.35)	(0.16)	(0.16)	(0.11)	(0.11)	(0.18)	(0.18)
Low parental education X Reform		1.26		0.60		1.74		2.24
		(0.62)		(0.64)		(0.80)		(3.67)
High parental education X Reform		1.03		0.85		0.18		2.27
		(0.99)		(1.89)		(0.19)		(6.06)
N	3,280	3,280	3,553	3,553	4,453	4,453	3,939	3,939

Notes: The coefficients are reported as odds ratios. Standard errors in parentheses. All models control for the survey (ESS or SHARE), survey wave of the ESS, a linear time trend, and interactions between the linear time trend and parental education (estimates for these controls are not shown).
Sources: ESS, Waves 1–9 and SHARE, Waves 1–7.
* $p < 0.05$ (two-tailed tests)

Table S6. Logistic Regression Models Estimating the Effects of the Reforms in the Minimum School Leaving Age on Post-Secondary Education (Non-Tertiary or Tertiary, ISCED1997 levels 4 to 6)

	**Austria**		**Denmark**		**France**		**Netherlands**	
	(1)	(2)	(3)	(4)	(5)	(6)	(7)	(8)
Low parental education	0.46*	0.47*	0.46*	0.43*	0.31*	0.32*	0.43*	0.44*
	(0.10)	(0.11)	(0.06)	(0.07)	(0.05)	(0.06)	(0.08)	(0.09)
High parental education	3.49*	3.39*	4.02*	3.66*	4.01*	4.24*	4.81*	5.19*
	(0.69)	(0.76)	(0.78)	(0.78)	(1.00)	(1.16)	(1.20)	(1.46)
Reform	1.15	1.14	1.11	1.32	0.70*	0.62	0.84	0.75
	(0.22)	(0.30)	(0.16)	(0.28)	(0.11)	(0.18)	(0.13)	(0.26)
Male	1.24*	1.24*	0.56*	0.56*	0.97	0.97	1.49*	1.49*
	(0.11)	(0.11)	(0.04)	(0.04)	(0.07)	(0.07)	(0.11)	(0.11)
Low parental education X Reform		1.13		0.75		1.17		1.10
		(0.51)		(0.24)		(0.41)		(0.44)
High parental education X Reform		0.87		0.66		1.31		1.38
		(0.42)		(0.26)		(0.71)		(0.77)
N	3,280	3,280	3,996	3,996	4,453	4,453	3,939	3,939

Notes: The coefficients are reported as odds ratios. Standard errors in parentheses. All models control for the survey (ESS or SHARE), survey wave of the ESS, a linear time trend, and interactions between the linear time trend and parental education (estimates for these controls are not shown).
Sources: ESS, Waves 1–9 and SHARE, Waves 1–7.
* $p < 0.05$ (two-tailed tests)

Table S7. Number of Respondents with Missing Information on Parental Education

	**Austria**	**Denmark**	**France**	**Netherlands**
European Social Survey	84	1,327	1,054	1,999
SHARE	1,359	1,088	2,315	1,422

Sources: ESS, Waves 1–9 and SHARE, Waves 1–7.

Table S8. OLS Regression Models Estimating the Effects of the Reforms in the Minimum School Leaving Age on Years of Education (Standardized within Each Country), Using Switzerland as Control Case

	**Austria**		**Denmark**		**France**		**Netherlands**	
	(1)	(2)	(3)	(4)	(5)	(6)	(7)	(8)
Low parental education	–0.55*	–0.54*	–0.47*	–0.46*	–0.54*	–0.55*	–0.45*	–0.47*
	(0.05)	(0.05)	(0.05)	(0.05)	(0.04)	(0.04)	(0.05)	(0.05)
High parental education	0.49*	0.51*	0.51*	0.51*	0.57*	0.57*	0.56*	0.55*
	(0.06)	(0.06)	(0.06)	(0.06)	(0.07)	(0.07)	(0.06)	(0.06)
Reform	–0.03	–0.08	–0.15*	–0.15*	0.10*	0.13*	0.05	0.14*
	(0.03)	(0.04)	(0.03)	(0.04)	(0.03)	(0.05)	(0.03)	(0.06)
Male	0.32*	0.32*	0.06*	0.06*	0.22*	0.22*	0.26*	0.26*
	(0.02)	(0.02)	(0.02)	(0.02)	(0.02)	(0.02)	(0.02)	(0.02)
Low parental education X Reform		0.08		0.03		–0.07		–0.13
		(0.07)		(0.07)		(0.06)		(0.08)
High parental education X Reform		0.14		–0.02		0.02		–0.10
		(0.08)		(0.08)		(0.09)		(0.10)
N	6,488	6,488	6,922	6,922	7,625	7,625	6,865	6,865

Notes: Standard errors in parentheses. All models control for the survey (ESS or SHARE), survey wave of the ESS, a linear time trend, and interactions between the linear time trend and parental education (estimates for these controls are not shown). Switzerland is used as a control group (reform = 0 for respondents who lived in Switzerland). The outcome is standardized within each country to account for variation in the distribution of educational attainment between Switzerland and the treatment countries (Austria, Denmark, France, and the Netherlands).
Sources: ESS, Waves 1–9 and SHARE, Waves 1–7.
* $p < 0.05$ (two-tailed tests)

Table S9. OLS Regression Models Estimating the Effects of the Reforms in the Minimum School Leaving Age on Years of Education, Separate Results for Women and Men

	All		Women		Men	
	(1)	(2)	(3)	(4)	(5)	(6)
			Panel A: Austria			
Low parental education	–1.67* (0.19)	–1.61* (0.22)	–1.71* (0.28)	–1.52* (0.32)	–1.64* (0.26)	–1.71* (0.29)
High parental education	1.24* (0.24)	1.19* (0.27)	1.35* (0.36)	1.39* (0.41)	1.05* (0.30)	0.88* (0.34)
Reform	0.17 (0.20)	0.11 (0.29)	0.17 (0.29)	–0.16 (0.43)	0.18 (0.26)	0.41 (0.38)
Male	0.67* (0.09)	0.67* (0.09)				
Low parental education X Reform		0.26 (0.44)		0.82 (0.65)		–0.27 (0.57)
High parental education X Reform		–0.26 (0.58)		0.23 (0.83)		–0.88 (0.79)
N	3,280	3,280	1,754	1,754	1,526	1,526
			Panel B: Denmark			
Low parental education	–1.18* (0.16)	–1.20* (0.18)	–1.05* (0.22)	–1.13* (0.25)	–1.32* (0.22)	–1.29* (0.25)
High parental education	1.41* (0.20)	1.32* (0.22)	1.49* (0.28)	1.42* (0.31)	1.36* (0.28)	1.26* (0.31)
Reform	0.07 (0.16)	0.20 (0.25)	–0.14 (0.23)	0.06 (0.34)	0.26 (0.23)	0.32 (0.35)
Male	–0.41* (0.07)	–0.41* (0.07)				

Low parental education X Reform		–0.11<br>(0.37)		–0.36<br>(0.52)		0.11<br>(0.52)
High parental education X Reform		–0.40<br>(0.42)		–0.33<br>(0.58)		–0.46<br>(0.61)
N	3,996	3,996	2,071	2,071	1,925	1,925
			Panel C: France			
Low parental education	–2.87*<br>(0.27)	–2.75*<br>(0.30)	–2.68*<br>(0.37)	–2.46*<br>(0.41)	–3.10*<br>(0.38)	–3.10*<br>(0.43)
High parental education	2.04*<br>(0.40)	1.99*<br>(0.43)	2.36*<br>(0.55)	2.21*<br>(0.61)	1.65*<br>(0.57)	1.72*<br>(0.62)
Reform	–0.30<br>(0.22)	–0.62<br>(0.48)	–0.23<br>(0.32)	–0.79<br>(0.69)	–0.37<br>(0.31)	–0.42<br>(0.68)
Male	0.37*<br>(0.10)	0.38*<br>(0.10)				
Low parental education X Reform		0.49<br>(0.55)		0.93<br>(0.79)		0.03<br>(0.78)
High parental education X Reform		–0.32<br>(0.88)		–0.88<br>(1.25)		0.33<br>(1.23)
N	4,453	4,453	2,389	2,389	2,064	2,064
			Panel D: Netherlands			
Low parental education	–1.30*<br>(0.20)	–1.29*<br>(0.23)	–1.53*<br>(0.27)	–1.56*<br>(0.30)	–1.06*<br>(0.31)	–0.99*<br>(0.35)
High parental education	1.61*<br>(0.27)	1.60*<br>(0.30)	1.69*<br>(0.35)	1.72*<br>(0.40)	1.50*<br>(0.40)	1.48*<br>(0.46)
Reform	–0.13<br>(0.16)	–0.17<br>(0.41)	0.03<br>(0.21)	0.09<br>(0.55)	–0.29<br>(0.25)	–0.49<br>(0.62)

Male	0.47*	0.47*				
	(0.08)	(0.08)				
Low parental education X Reform		0.06		–0.10		0.30
		(0.45)		(0.60)		(0.68)
High parental education X Reform		–0.03		0.11		–0.14
		(0.61)		(0.81)		(0.93)
N	3,939	3,939	2,126	2,126	1,813	1,813

Notes: Standard errors in parentheses. All models control for the survey (ESS or SHARE), survey wave of the ESS, a linear time trend, and interactions between the linear time trend and parental education (estimates for these controls are not shown).
Sources: ESS, Waves 1–9 and SHARE, Waves 1–7.
* $p < 0.05$ (two-tailed tests)

FIGURES

Figure S1. Variation in Years of Education by Year of Birth

Panel A: Austria

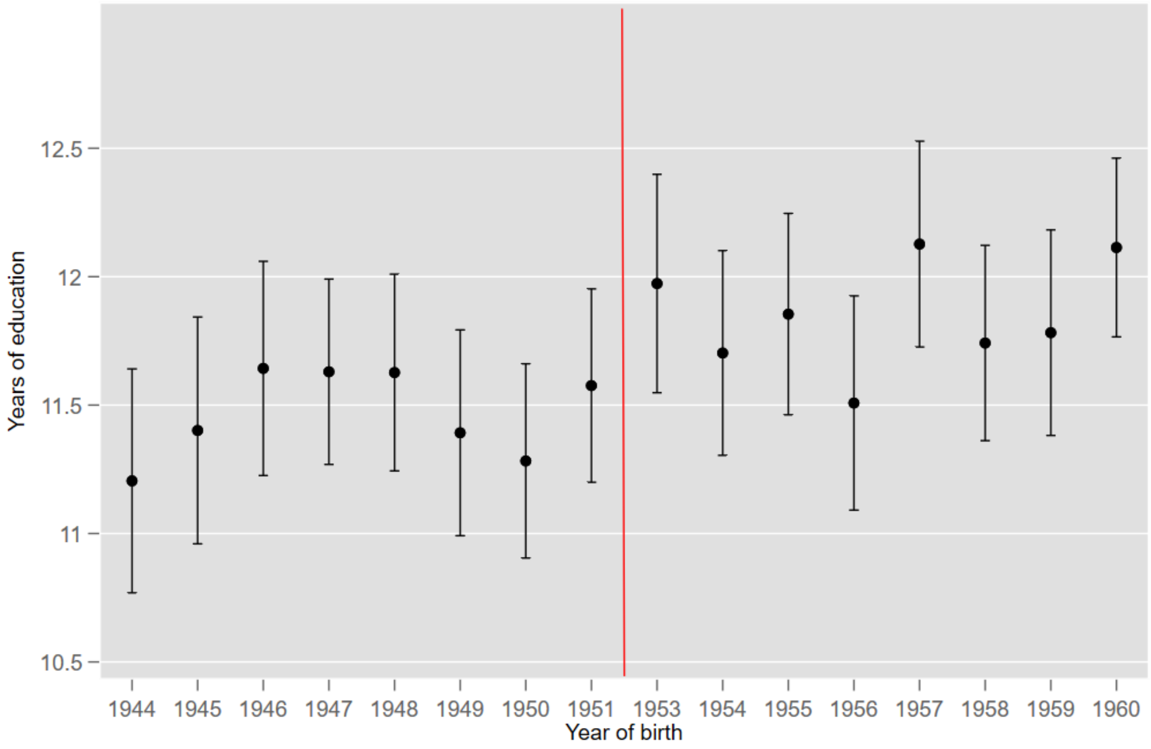

Notes: These estimates are obtained via a regression model with years of education as the outcome and a set of dummy variables of year of birth and a dummy of male as independent variables. The red line indicates the year of the reform in the minimum school leaving age (first birth year affected by the reform is omitted from the analysis, as discussed in the manuscript.)

Panel B: Denmark

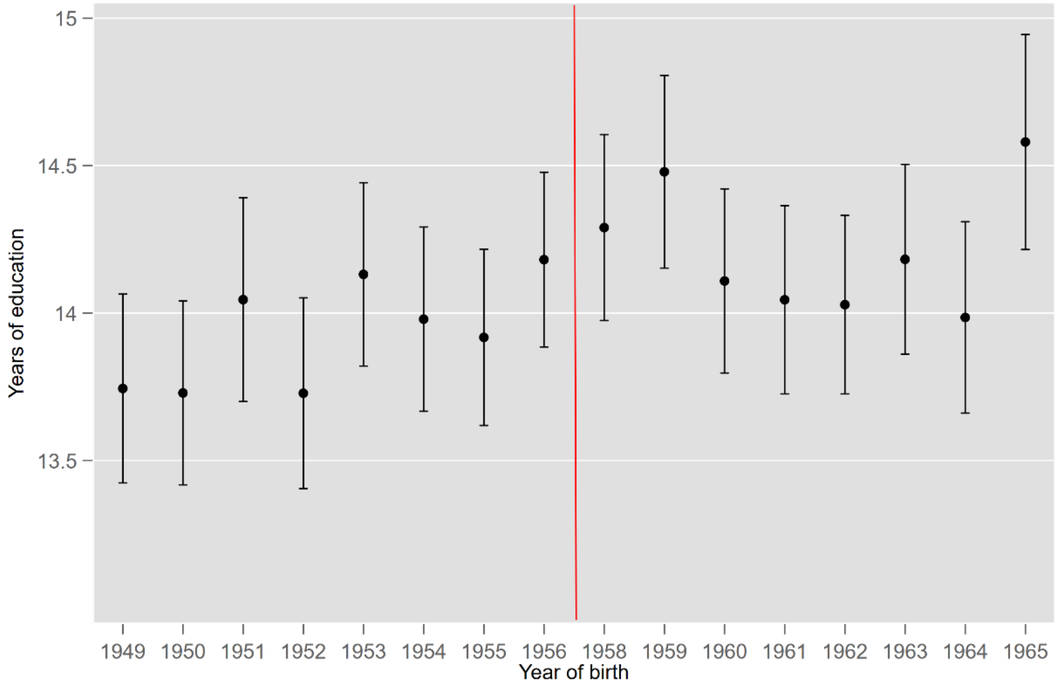

Notes: These estimates are obtained via a regression model with years of education as the outcome and a set of dummy variables of year of birth and a dummy of male as independent variables. The red line indicates the year of the reform in the minimum school leaving age (first birth year affected by the reform is omitted from the analysis, as discussed in the manuscript.)

Panel C: France

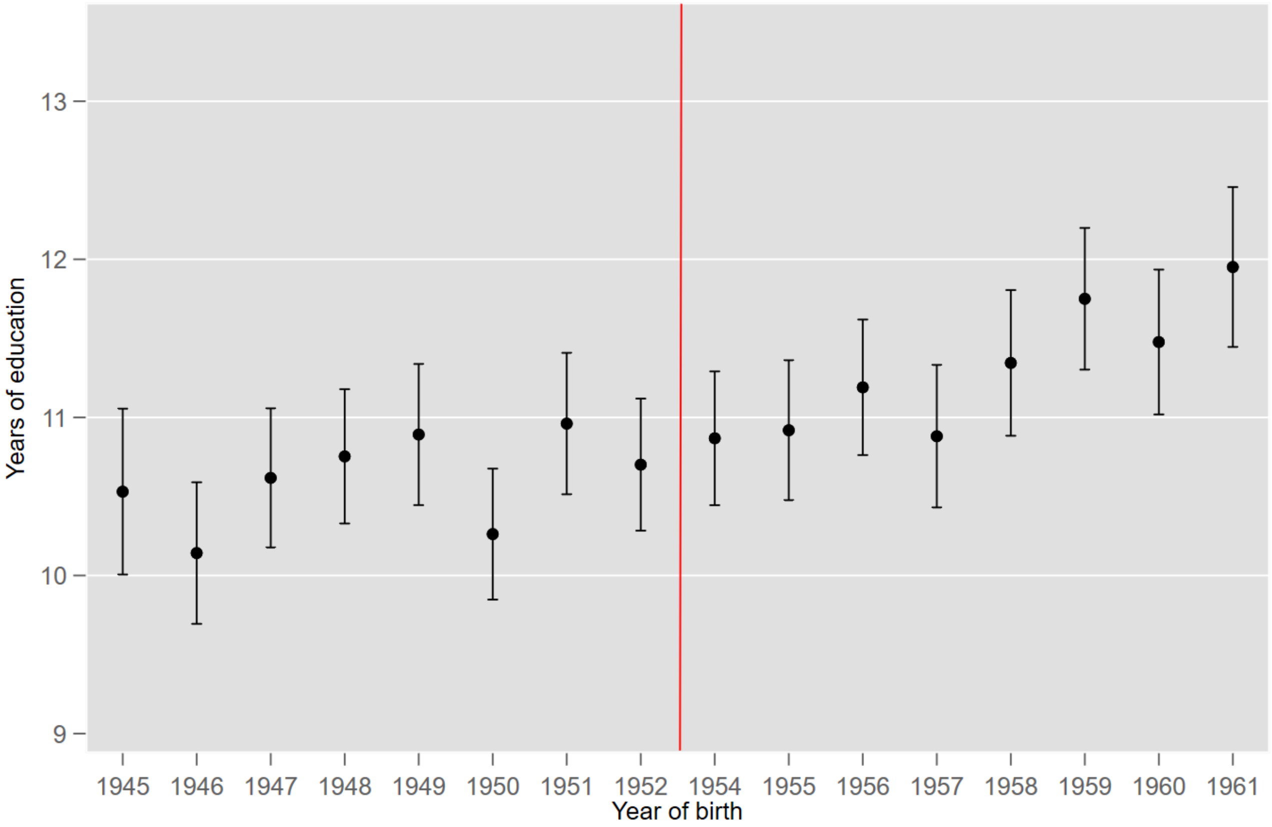

Notes: These estimates are obtained via a regression model with years of education as the outcome and a set of dummy variables of year of birth and a dummy of male as independent variables. The red line indicates the year of the reform in the minimum school leaving age (first birth year affected by the reform is omitted from the analysis, as discussed in the manuscript.)

Panel D: Netherlands:

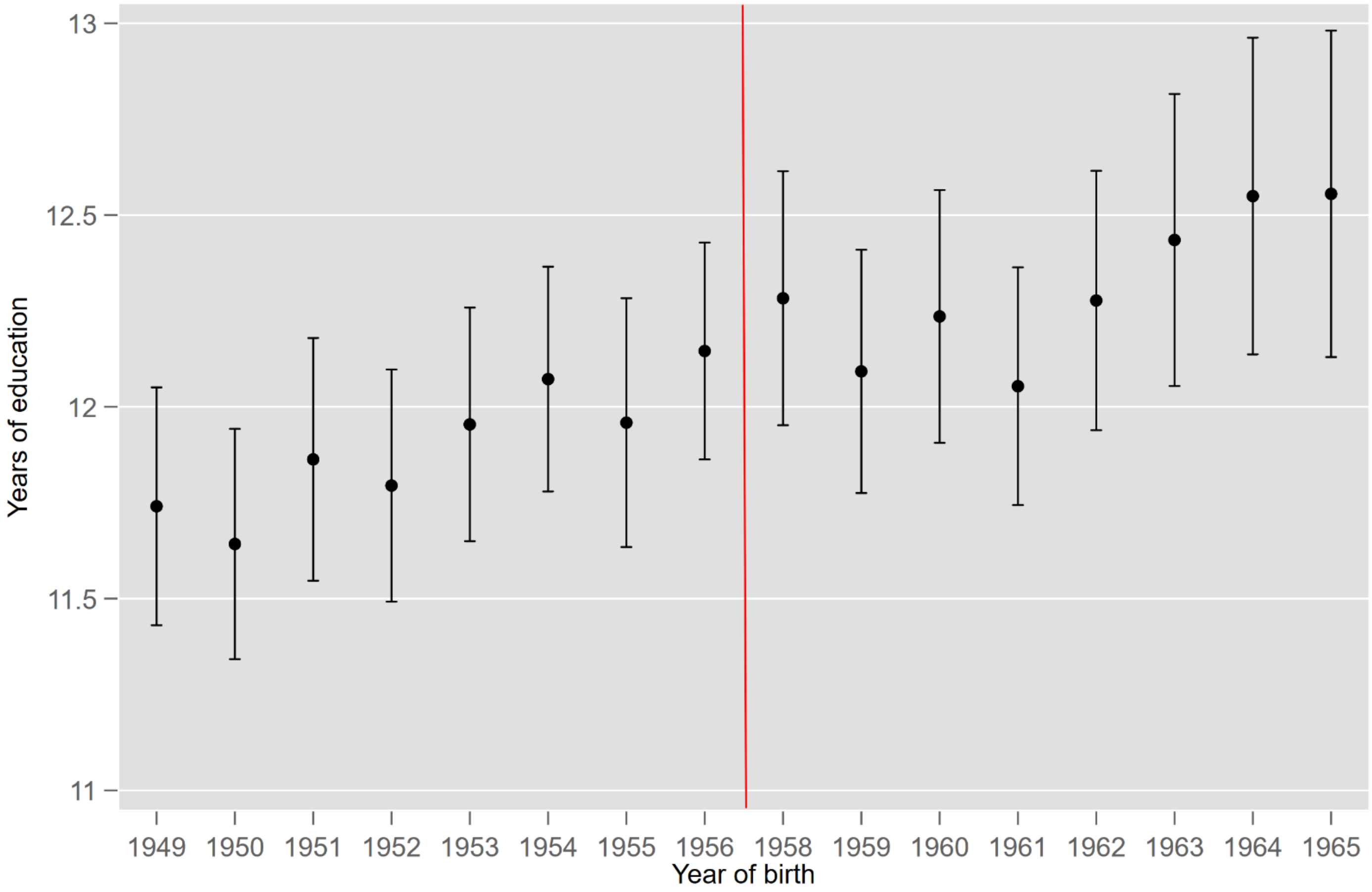

Notes: These estimates are obtained via a regression model with years of education as the outcome and a set of dummy variables of year of birth and a dummy of male as independent variables. The red line indicates the year of the reform in the minimum school leaving age (first birth year affected by the reform is omitted from the analysis, as discussed in the manuscript.)

Figure S2. Variation in Years of Education by Year of Birth by Parental Education

Panel A: Austria

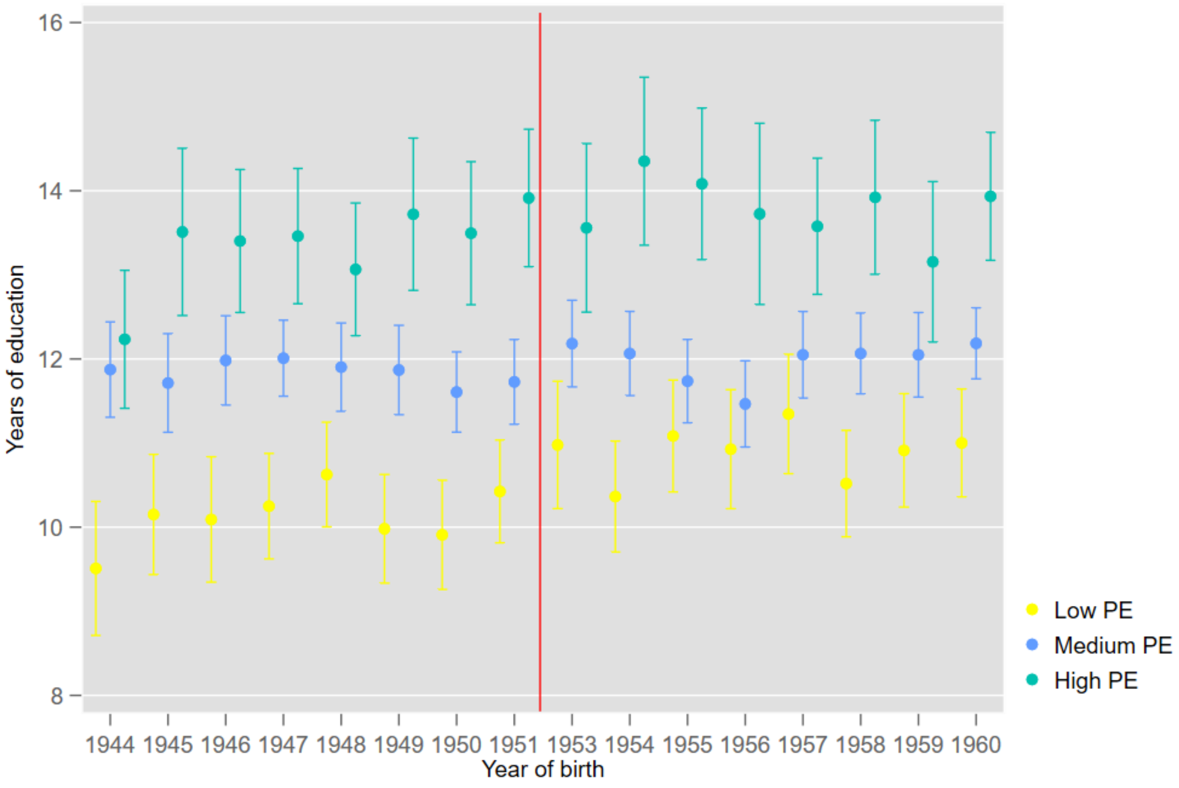

Notes: These estimates are obtained via regression models with years of education as the outcome and a set of dummy variables of year of birth and a dummy of male as independent variables. Separate models are estimated for each level of parental education. The red line indicates the year of the reform in the minimum school leaving age (first birth year affected by the reform is omitted from the analysis, as discussed in the manuscript.)

Panel B: Denmark

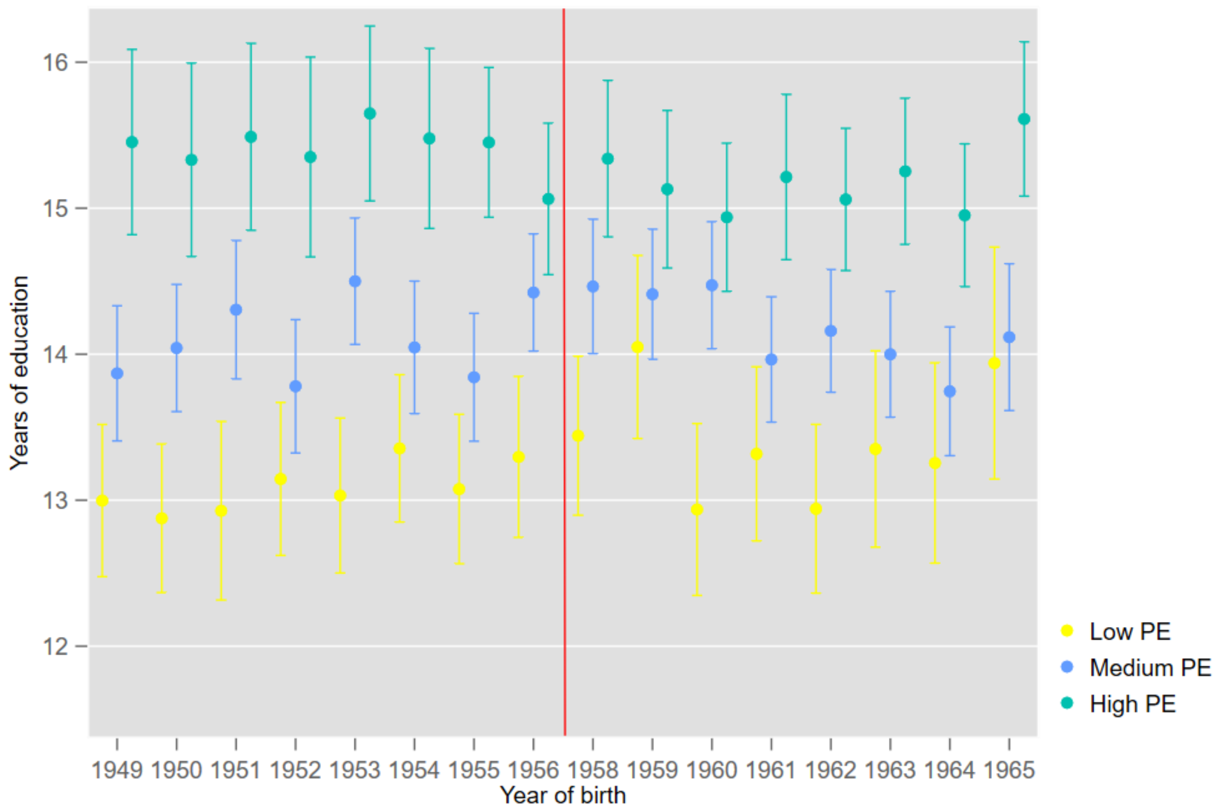

Notes: These estimates are obtained via regression models with years of education as the outcome and a set of dummy variables of year of birth and a dummy of male as independent variables. Separate models are estimated for each level of parental education. The red line indicates the year of the reform in the minimum school leaving age (first birth year affected by the reform is omitted from the analysis, as discussed in the manuscript.)

Panel C: France

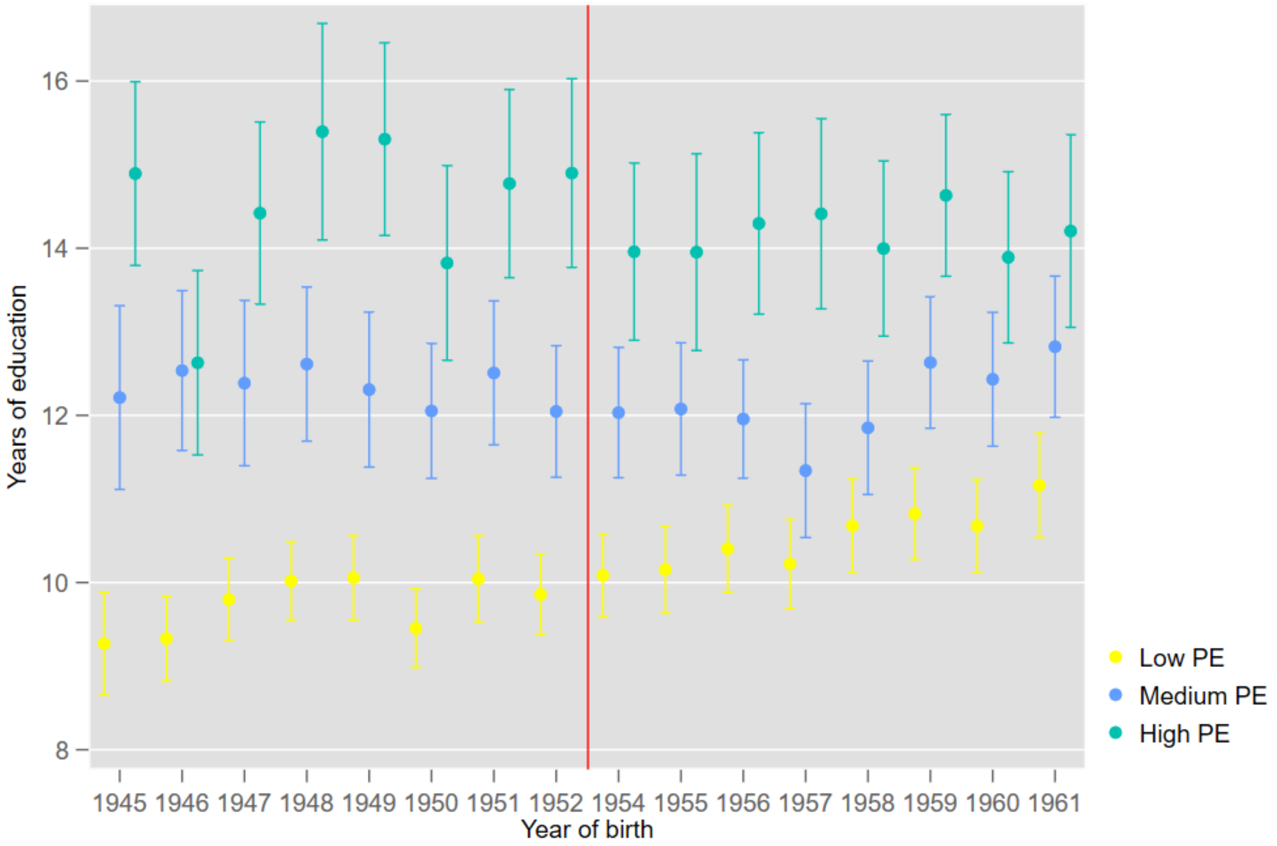

Notes: These estimates are obtained via regression models with years of education as the outcome and a set of dummy variables of year of birth and a dummy of male as independent variables. Separate models are estimated for each level of parental education. The red line indicates the year of the reform in the minimum school leaving age (first birth year affected by the reform is omitted from the analysis, as discussed in the manuscript.)

Panel D: Netherlands

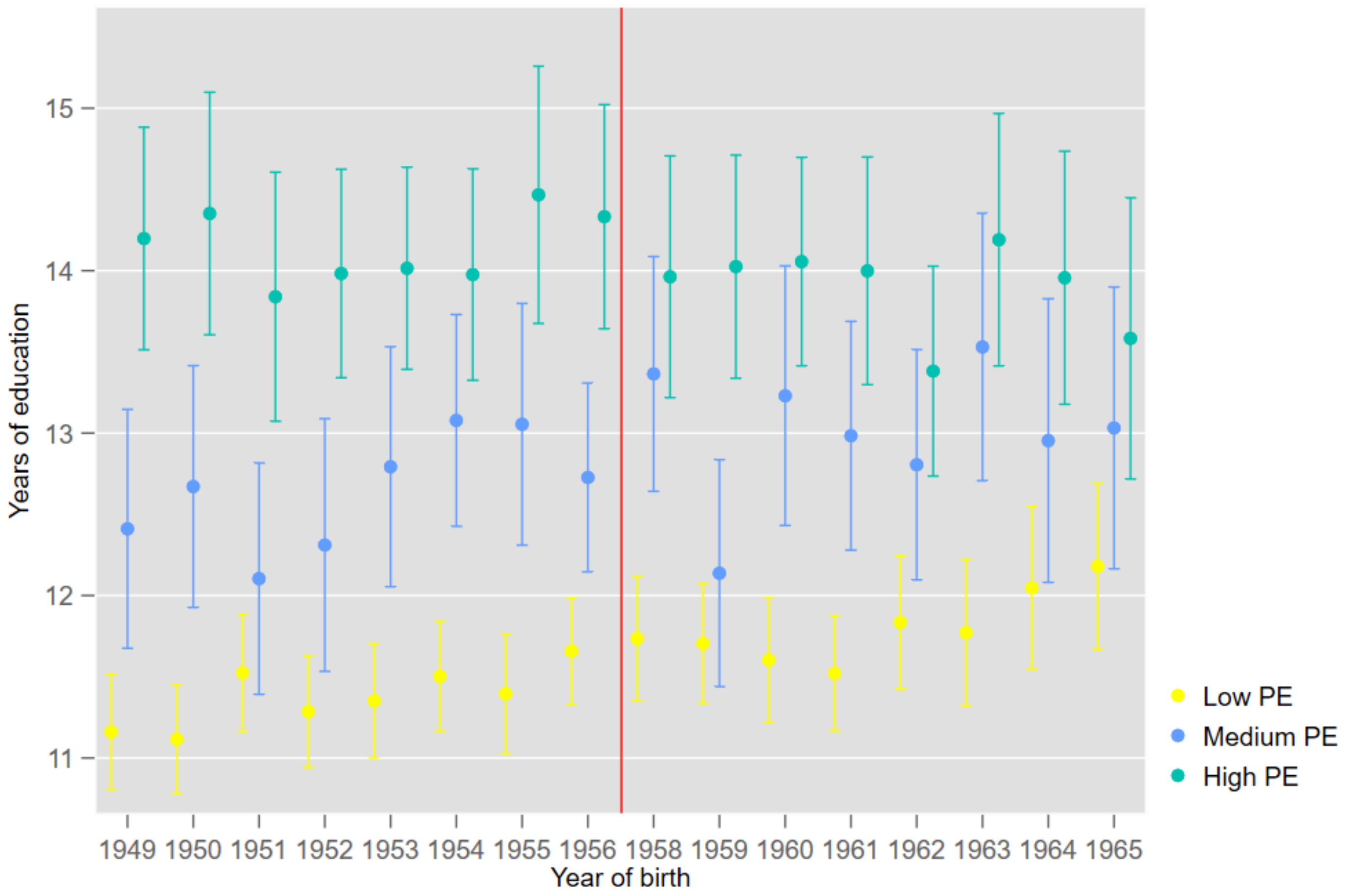

Notes: These estimates are obtained via regression models with years of education as the outcome and a set of dummy variables of year of birth and a dummy of male as independent variables. Separate models are estimated for each level of parental education. The red line indicates the year of the reform in the minimum school leaving age (first birth year affected by the reform is omitted from the analysis, as discussed in the manuscript.)
